# Past antihypertensive medication use is associated with lower levels of small vessel disease and lower Aβ plaque stage in the brains of older individuals

**DOI:** 10.1111/nan.12922

**Published:** 2023-08-01

**Authors:** Andrew J. Affleck, Perminder S. Sachdev, Glenda M. Halliday

**Affiliations:** ^1^ Neuroscience Research Australia (NeuRA) Sydney Australia; ^2^ Centre for Health Brain Ageing (CHeBA), Discipline of Psychiatry and Mental Health, Faculty of Medicine University of New South Wales Sydney Australia; ^3^ Neuropsychiatric Institute The Prince of Wales Hospital Sydney Australia; ^4^ School of Medical Sciences, Faculty of Medicine University of New South Wales Sydney Australia; ^5^ Brain and Mind Centre & Faculty of Medicine and Health School of Medical Sciences University of Sydney Sydney Australia

**Keywords:** Alzheimer's disease, antihypertensive medication, cerebrovascular disease, neuropathology, small vessel disease

## Abstract

**Aims:**

This study assesses the association of antihypertensive medication use on the severities of neuropathological cerebrovascular disease (CVD excluding lobar infarction) in older individuals.

**Methods:**

Clinical and neuropathological data were retrieved for 149 autopsy cases >75 years old with or without CVD or Alzheimer's disease and no other neuropathological diagnoses. Clinical data included hypertension status, hypertension diagnosis, antihypertensive medication use, antihypertensive medication dose (where available) and clinical dementia rating (CDR). Neuropathological CVD severity was evaluated for differences with anti‐hypertensive medication usage.

**Results:**

Antihypertensive medication use was associated with less severe white matter small vessel disease (SVD, mainly perivascular dilatation and rarefaction), with a 5.6–14.4 times greater likelihood of less severe SVD if medicated. No significant relationship was detected between infarction (presence, type, number and size), lacunes or cerebral amyloid angiopathy and antihypertensive medication use. Only increased white matter rarefaction/oedema and not perivascular dilation was associated with Alzheimer's pathology, with a 4.3 times greater likelihood of reduced Aβ progression through the brain if white matter rarefaction severity was none or mild. Antihypertensive medication use was associated with reduced Aβ progression but only in those with moderate to severe white matter SVD.

**Conclusions:**

This histopathological study provides further evidence that antihypertensive medication use in older individuals is associated with white matter SVD and not with other CVD pathologies. This is mainly due to a reduction in white matter perivascular dilation and rarefaction/oedema. Even in those with moderate to severe white matter SVD, antihypertensive medication use reduced rarefaction and Aβ propagation through the brain.

## Graphical abstract

Of common cerebrovascular pathologies, antihypertensive medication use in later life reduced pathological indices of small vessel disease in the white matter (mainly perivascular dilatation and rarefaction/oedema) with a greater likelihood (5.6–14.4 times) of less severe small vessel disease at autopsy if medicated. Less white matter rarefaction/oedema (not perivascular dilatation) was associated with lower Ab plaque scores at autopsy. In those with moderate or severe small vessel disease, antihypertensive medication use was associated with reduced CDR and Ab plaque scores.

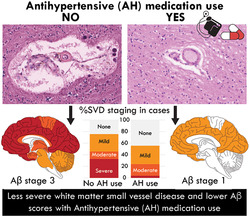

**Key Points**

- Of common cerebrovascular pathologies, only pathological indices of small vessel disease in the white matter (mainly perivascular dilatation and rarefaction/oedema) were associated with antihypertensive medication use in later life.
- Antihypertensive medication use was a significant predictor of the severity of white matter small vessel disease—there was a much greater likelihood (5.6–14.4 times) of less severe white matter small vessel disease at autopsy if medicated.
- White matter rarefaction/oedema and not perivascular dilatation was associated with Aβ plaque scores at autopsy.
- In those with moderate or severe white matter small vessel disease, antihypertensive medication use associated with reduced CDR and Aβ plaque scores.

## INTRODUCTION

Blood pressure‐lowering antihypertensive medications are used to reduce the substantial risk of cardiovascular events and death due to hypertension [1]. These include angiotensin receptor blockers, angiotensin‐converting enzyme inhibitors (ACEIs), beta‐blockers, calcium channel blockers and diuretics, which act on reducing vasodilation (angiotensin receptor blockers, ACEIs and calcium channel blockers), sympathetic tone (beta blockers), cardiac output (beta blockers and calcium channel blockers) and renin secretion (beta blockers) and increasing diuresis (diuretics) [2]. These medications are used to reduce arterial hypertension in different patient populations from >160/100 mmHg (systolic/diastolic) to <140/90 mmHg [3].

In the brain, hypertension is associated with reduced perfusion due to structural and functional alterations in cerebral vessels that predispose the brain to white matter damage, atrophy, macro‐ and microbleeds, ischaemia and the deposition of pathologic proteins [4]. A recent meta‐analysis of five large clinical trials has shown that blood pressure‐lowering antihypertensive medication use over <5 years in late–mid and later life reduces the risk of incident dementia, and the greater the fall in blood pressure, the greater the reduction in dementia risk [5]. However, the type of dementia has not been confirmed pathologically in clinical trials to date, and increased vascular risk is required to enter these trials, making it highly likely that vascular pathology is a contributor to the dementia [5].

Cerebrovascular disease (CVD) pathologies have been difficult to measure accurately, as identified most recently [6] and also previously [7]. Using a Delphi method [7], a number of CVD pathologies were defined and identified as relevant for cognitive impairment, including cerebral amyloid angiopathy (CAA), large infarcts, lacunar infarcts, microinfarcts, arteriolosclerosis, perivascular space dilation and myelin loss or rarefaction. Brain arteriolosclerosis (pathologic arteriolar wall thickening) has been more recently defined but is found in more than 80% of autopsies over the age of 80 [8]. While Alzheimer's disease (AD) and most CVD pathologies (but not CAA) are largely uncorrelated [6, 9], brain arteriosclerosis and infarcts are related to the onset and severity of AD dementia, suggesting an additive effect in the presence of AD pathologies [9]. Importantly, in the absence of significant age‐related neurodegenerative diseases, microvascular brain damage in the form of perivascular space dilation and rarefaction has been found to be the chief substrate of vascular dementia [10].

It is now clear that potentially preventable CVD pathologies are more prevalent than AD pathologies (amyloid and neuritic plaques and neurofibrillary tangles [NFT]) in pathological cohorts of dementia (53% have either only pure CVD or AD + CVD compared to just 24% with pure AD) [11, 12] with limited evidence that antihypertensive medication use prevents dementia in those without CVD [13]. A systematic review on hypertension and AD neuropathological change found a modest positive association between hypertension and AD neuropathology [14], but we [15] and others [16] have observed no change in the amount of AD‐related pathology in predilection sites following antihypertensive medication use. In our study, antihypertensive medication use was associated with less progression of AD pathology (amyloid and neuritic plaques and NFT scores) through the brain [15], suggesting that a vascular mechanism may be involved.

Neuroimaging studies have consistently shown that antihypertensive medication use protects against white matter damage but not brain atrophy or stroke [17, 18, 19, 20] and that the size of this effect relates to the magnitude of intensive blood pressure control [21]. Importantly, this is not due to histologically confirmed vessel wall thickness or atherosclerotic plaque [22] as brain arteriolosclerosis is present in more than 80% of autopsied individuals over 80 years of age [8]. A recent systematic review comparing neuroimaging with histopathology identified that radiological white matter lesions commonly included myelin pallor, rarefaction/oedema and perivascular space dilatation [23]. As the effects of antihypertensive medication use on these histopathological measures of white matter damage have not been performed, we investigated CVD pathologies in the brain tissue of individuals who did and did not take antihypertensive medications during later life to determine the effect of antihypertensive medication use on such neuropathology.

## MATERIALS AND METHODS

### Case selection

All cases were sourced from the human brain collection held at the Sydney Brain Bank, which works with regional brain donor programmes in Sydney, Australia, to follow participants, longitudinally gathering research and clinical data during life. Brain tissue was retrieved as soon as possible after death with half of the brain processed and stored frozen at −80°C and the other half placed into 15% formalin fixative for 2 weeks for histological assessment. When this type of processing was not suitable, the whole brain was placed into 15% formalin fixative for 2 weeks. Neuropathological case characterisation was in accordance with the current consensus criteria [7, 24]. This included assessing the progression of Aβ deposition in the brain using the A score (A0 no Aβ, A1 neocortical Aβ, A2 subcortical Aβ, A3 brainstem Aβ [24]) and the progression of NFTs in the brain using the B score (B0 no NFT, B1 transentorhinal NFT, B2 limbic NFT, B3 neocortical NFT [24]). The Sydney Brain Bank and the regional brain donor programmes all hold appropriate ethics approval from their respective institutional Human Research Ethics Committees and all participants and/or their senior available next of kin consented to brain retrieval and the use of clinical and research data for future research studies. This particular study was approved by the University of New South Wales Human Research Ethics Committee (approval no. HC15613), and the cases were previously assessed for the effect of antihypertensive medication use on AD pathology [15].

A total of 149 cases were selected based on the dominant type of diagnostic neuropathology present; being either no significant neuropathology (incidental, non‐diagnostic pathologies allowed) or CVD with or without AD neuropathologic change and no other pathological diagnoses [7, 24] (see Table 1). CVD included all cases except those with lobar infarction equating to > 50 mL of tissue loss [25] as current pathological criteria for AD require the systematised sampling of multiple lobar regions [24]. Those with incomplete clinical data, especially hypertension status and antihypertensive medication usage (see Figures S1 and S2 for further breakdown) and those aged at death <75 years old (median age at death in Australia for both men and women is 85–89 years old; see https://www.aihw.gov.au/reports/life-expectancy-death/deaths-in-australia/contents/age-at-death) were also excluded to further reduce the potential for neuropathologies other than CVD or AD (dementia and CVD are the second and third most prevalent causes of death in Australians >75 years of age at death; see https://www.aihw.gov.au/reports/life-expectancy-death/deaths-in-australia/contents/leading-causes-of-death).

**TABLE 1:** Case demographics and clinical data—entire cohort.

Group (*n*)	AH medicated			Hypertensive			AD neuropathologic change	
	Yes (79)	No (70)	Statistics	Yes (73)	No (76)	Statistics	Not/low (53)	Intermediate/high (96)	Statistics^c^
Age at death, mean years (SD)	**89 (5.5)**	**86 (5.8)**	***t*(147) = −2.4** ***p* = 0.02** **CI [−4.1, −0.3]**	88 (5.6)	87 (5.7)	*t*(147) = 1.8 *p* = 0.072 CI [−0.09, 3.5]	**89** **(6.1)**	**87** **(5.4)**	***t*(147) = 2.1** ***p* = 0.039** **CI [0.23, 3.8]**
Female, *n* (%)	48 (61%)	41 (59%)	𝜒^2^(1) = 0.07 *p* = 0.786	46 (63%)	43 (57%)	𝜒^2^(1) = 0.64 *p* = 0.423	**26** **(49%)**	**63** **(66%)**	**𝜒** ^**2**^ **(1) = 3.9** ***p* = 0.048**
CDR 0 or 0.5, *n* (%)	**42 (53%)**	**18 (26%)**	***𝜒*** ^**2**^ **(1) = 11.6** ***p* = 0.001**	33 (45%)	27 (36%)	𝜒^2^(1) = 1.5, *p* = 0.228	**34** **(64%)**	**26** **(27%)**	***𝜒*** ^**2**^ **(1) = 19.5** ***p* < 0.001**
PMD, mean hours (SD)	21 (17.3)	26 (19.7)	*t*(147) = 1.6 *p* = 0.102 CI [−0.77, 10.47]	21 (15.5)	24 (21.0)	*t*(147) = −1.1 *p* = 0.294 CI [−9.0, 2.6]	25 (19.0)	22 (18.3)	*t*(147) = 0.96 *p* = 0.336 CI [−2.7, 8.8]
Hypertensive, *n* (%)	**57 (72%)**	**16 (23%)**	**𝜒** ^**2**^ **(1) = 36.1** ***p* < 0.001**	‐	‐	‐	27 (51%)	46 (48%)	𝜒^2^(1) = 0.13 *p* = 0.723
Hypertension duration, mean years (SD)^a^	13 (9.3)	13 (11.5)	*t*(64) = 0.02 *p* = 0.988 CI [−5.9, 6.6]	13 (9.7)	‐	‐	14 (9.6)	13 (9.9)	*t*(64) = 0.64 *p* = 0.526 CI [−3.1, 6.8]
VCING criteria									
Low, *n* (%)	42 (53%)	29 (41%)	𝜒^2^(2) = 3.3 *p* = 0.193	41 (56%)	30 (39%)	𝜒^2^(2) = 4.3 *p* = 0.117	23 (43%)	48 (50%)	𝜒^2^(2) = 2.9 *p* = 0.230
Moderate, *n* (%)	20 (25%)	17 (24%)		16 (22%)	21 (28%)		11 (21%)	26 (27%)	
High, *n* (%)	17 (22%)	24 (34%)		16 (22%)	25 (33%)		19 (36%)	22 (23%)	
Apolipoprotein E^b^ (*N* = 94), *n* (%)	56 (60%)	38 (40%)	N/A	51 (54%)	43 (46%)	N/A	31 (33%)	63 (67%)	N/A
Apolipoprotein E *ε4* carrier, *n* (% of column metric)	16 (29%)	18 (47%)	𝜒^2^(1) = 3.5 *p* = 0.063	18 (35%)	16 (37%)	𝜒^2^(1) = 0.04 *p* = 0.847	**3** **(10%)**	**31** **(49%)**	𝜒^2^(1) = 14.1 *p* = **<0.001**

*Notes*: Hypertensive cases based on clinical assessment. Statistically significant results (*p* < 0.05) are in bold.

Abbreviations: N, number of cases, PMD, post‐mortem delay; SD, standard deviation.

^a^ Average duration in years of hypertensive cases. Seven cases did not have hypertension duration data due to incomplete clinical records (i.e. did have confirmation of hypertensive status but no information on the date of diagnosis).

^b^ ApoE data not available on the entire cohort, was available on 94 cases.

^c^ Independent samples *t*‐tests were used for quantitative variables with the addition of bootstrapping to validate and calculate robust confidence intervals, chi‐square tests were used for qualitative variables.

### Clinical data assessed

Clinical data were retrieved from integrated clinical and autopsy databases as well as a retrospective review of donor clinical files that included general practitioner/specialist medical records and participant and/or family questionnaires as well as self‐reports. Details on hypertension status, hypertension diagnosis, antihypertensive medication use, antihypertensive medication dose and clinical dementia rating (CDR) [26] were obtained. Antihypertensive medications were cross‐referenced with the Monthly Index of Medical Specialities (MIMS) online database (available at https://www.mimsonline.com.au) to confirm major antihypertensive medication classes. These included angiotensin receptor blockers, ACEIs, beta‐blockers, calcium channel blockers and diuretics. All other medications that were prescribed were also noted, cross‐referenced via MIMS and placed into overarching medication classes. In addition to cases taking the antihypertensive medications already described (*n* = 79), a further five other medication classes were identified to be vascular‐acting agents and taken by the cases (see Table S1). These included medications for; angina (*n* = 24), hyperlipidaemia (*n* = 21), cardiac inotropy (*n* = 17), hypoglycaemia (*n* = 6) and arrhythmia (*n* = 5) agents. Considering that the majority of the cohort had taken antihypertensive medications (53%) and that the largest group of other vascular‐acting agents was <30% of the total antihypertensive medicated group and only 16% of the overall cohort, we have concentrated on the differences in the antihypertensive versus no medication groups with data relating to other vascular acting agents given in the Supporting Information (Tables S1 and S2).

The ε4 allele of the *apolipoprotein E* (*ApoE*) gene is regarded as the strongest genetic risk factor for sporadic AD as well as being a risk factor for cardiovascular disease [27], and this was reflected in our cohort of older individuals where only three ε4 carriers did not have AD, two of whom were medicated for hypertension (see table 1). In terms of CVD, ε2 carriers have an increased risk of gross haemorrhage and were therefore not represented in our cohort, while ε4 carriers have more severe CAA often in association with AD pathology [28]. In those with *ApoE* genotype data (63%), 64% were ε3 carriers, 23% had one ε4 allele, and 13% were homozygous for the ε4 allele. There were 14 ε4 carriers that had infarct(s) (six on antihypertensive medications; see Table 2) and 11 who had small vessel disease (SVD) (three on antihypertensive medications; see Table 3).

**TABLE 2:** Infarcts.

Any type non‐lobar infarct (*n*)	Present (77)	Absent (72)	Statistics^c^
**AD intermediate/high level change, *n* (%)**	**42 (55%)**	**54 (75%)**	**𝜒** ^**2**^ **(1) = 6.8** ***p* = 0.009**
Age at death, mean years (SD)	88 (5.5)	87 (6.0)	*t*(147) = −0.34 *p* = 0.725 CI [−2.1, 1.4]
**Female, *n* (%)**	**35 (45%)**	**54 (75%)**	**𝜒** ^**2**^ **(1) = 13.5** ***p* = <0.001**
PMD, mean hours (SD)	23 (20.3)	23 (16.6)	*t*(147) = −0.03 *p* = 0.978 CI [−5.5, 5.2]
AH medicated, *n* (%)	41 (53%)	38 (53%)	𝜒^2^(1) = 0.003 *p* = 0.954
Hypertensive, *n* (%)	38 (49%)	35 (49%)	𝜒^2^(1) = 0.008 *p* = 0.928
Hypertension duration, mean years (SD) [*n*]^a^	13 (7.9) [36]	14 (11.7) [30]	*t*(64) = 0.23 *p* = 0.827 CI [−3.9, 5.2]
Apolipoprotein E^b^ (*N* = 94), *n* (%)	42 (45%)	52 (55%)	N/A
Apolipoprotein E *ε4* carrier, *n* (% of infarct presence or absence)	14 (33%)	20 (39%)	𝜒^2^(1) = 0.68 *p* = 0.410

*Notes*: Hypertensive cases based on clinical assessment. Statistically significant results (*p* < 0.05) are in bold.

Abbreviations: N, number of cases, PMD, post‐mortem delay; SD, standard deviation.

^a^ Average duration in years of hypertensive cases. Seven cases did not have hypertension duration data due to incomplete clinical records (i.e. did have confirmation of hypertensive status but no information on the date of diagnosis).

^b^ ApoE data not available on the entire cohort, was available on 94 cases.

^c^ Independent samples *t*‐tests were used for quantitative variables with the addition of bootstrapping to validate and calculate robust confidence intervals, chi‐square tests were used for qualitative variables.

**TABLE 3:** Small vessel disease.

	Small vessel disease, severity (*n*)				Statistics^c^
	None (52)	Mild (46)	Moderate (25)	Severe (26)	
Age at death, mean years (SD)	89 (6.3)	87 (5.3)	87 (5.2)	86 (5.3)	*F*(3, 145) = 2.1 *p* = 0.108
Female, *n* (%)	35 (67%)	25 (54%)	15 (60%)	14 (54%)	𝜒^2^(3) = 2.2 *p* = 0.538
CDR 0 or 0.5, *n* (%)	25 (48%)	19 (41%)	10 (40%)	6 (23%)	𝜒^2^ (3) = 4.5 *p* = 0.209
PMD, mean hours (SD)	22 (21.2)	22 (18.5)	22 (15.0)	28 (16.1)	*F*(3, 145) = 0.704 *p* = 0.551
AH Medicated, *n* (%)	**35 (67%)**	**25 (54%)**	**16 (64%)**	**3 (12%)**	**𝜒** ^**2**^ **(3) = 23.5** ***p* < 0.001**
Hypertensive, *n* (%)	**26 (50%)**	**27 (59%)**	**14 (56%)**	**6 (23%)**	**𝜒** ^**2**^ **(3) = 9.2** ***p* =** 0**.026**
Hypertension duration^a^, mean years (SD) (*n*)	13 (9.3) [23]	15 (11.3) [25]	10 (8.5) [12]	10 (4.6) [6]	*F*(3, 62) = 1.05 *p* = 0.375
AD intermediate/high level change, *n* (%)	31 (60%)	29 (63%)	17 (68%)	19 (73%)	𝜒^2^(3) = 1.6 *p* = 0.670
Apolipoprotein E^b^ (*N* = 94), *n* (% of total ApoE)	33 (35%)	36 (38%)	16 (17%)	9 (10%)	N/A
Apolipoprotein E *ε4* carrier, *n* (% of SVD severity)	10 (30%)	13 (36%)	5 (31%)	6 (67%)	𝜒^2^(3) = 4.3 *p* = 0.232

*Notes*: Hypertensive cases based on clinical assessment. Statistically significant results (*p* < 0.05) are in bold.

Abbreviations: N, number of cases, PMD, post‐mortem delay; SD, standard deviation.

^a^ Average duration in years of hypertensive cases. Seven cases did not have hypertension duration data due to incomplete clinical records (i.e. did have confirmation of hypertensive status but no information on the date of diagnosis).

^b^ ApoE data not available on the entire cohort, was available on 94 cases.

^c^ One‐way ANOVA was used for quantitative variables, chi‐square tests were used for qualitative variables.

### Neuropathology data assessed

Gross CVD pathologies were assessed on coronal slices of both the freshly dissected hemisphere (sliced at 1 cm) and the formalin‐fixed brain tissue (cut on a rotary slicer at 3 mm). This gave available gross infarct data, which included brain region involvement, size of infarct (as either ‘small’ or ‘large’), number of infarct/s (single or multiple) and type of tissue involvement (grey matter, white matter or both). These data were available for all cases; however, not all the cases had specific measurement data for infarct involvement. As a result, 20–25 of the most recent cases that had specific infarct measurements were selected, and exact infarct dimensions were extracted (in the x, y and z planes) with an ellipsoid volume of infarct calculated for each case in that subset. The ellipsoid was selected as a more realistic three‐dimensional (3D) shape to approximate infarct volume, with other 3D shapes such as a sphere likely to underestimate and a cube or rectangular prism likely to overestimate infarct volume. Averages of these volumes were calculated based on broad brain region (cortical, subcortical or brainstem) and whether or not they were subjectively categorised as ‘large’ or ‘small’ (dichotomised as < or > 5000 mm^3^ or 5 mL). Brain regions that had multiple infarcts in the same region(s) were counted, and average multipliers were calculated. The resulting average ellipsoidal volumes were applied to the rest of the cases to obtain approximate infarct volumes for each case that had recorded infarcts. Lacunes were also assessed semi‐quantitatively as either none, few small or many small and/or large lacunes present.

From the fixed tissue slices, standardised tissue blocks were dissected cut at 10 μm and stained with Aβ antibody (1:500, 6F/3D clone, M0872, Dako, Glostrup, Denmark) using a standardised protocol [29]. CAA was assessed and recorded as either present or absent defined by either the involvement of parenchymal, meningeal and/or capillary CAA involvement as described in Love et al [30]. AD pathologies assessed have been previously reported [15].

### Measurement of SVD pathologies

As recently documented, there is no universally agreed‐upon method for categorising SVD pathologic changes [8]. The same standardised regions [29] were cut at 10 μm and stained with haematoxylin and eosin and for the present study, CVD pathology was assessed as outlined in Skrobot et al [7] and by applying Esiri et al's technique [10] of a semi‐quantitative score of none, mild, moderate and severe SVD. This incorporates progression and severities of a combination of relevant pathologies such as perivascular spaces, hyaline thickening of arteriolar walls, perivascular myelin pallor, cell and nerve fibre loss and white and grey matter gliosis (see Figure 2A for how this was operationalised).

A large subset of the cases (*n* = 118) was further analysed to measure the two main pathologies that contribute to the SVD severity score, namely, perivascular space (PVS) and rarefaction (RR) in the frontal white matter. To measure PVS, the haematoxylin and eosin‐stained slides from the superior frontal lobe of each case were scanned using an Aperio XT slide scanner. Whole slide scanned images were visualised and analysed using QuPath quantitative pathology and bioimage analysis software [31]. The white matter area was annotated, and a simple thresholder operator was applied. To normalise measurements, the PVS area was expressed as a percentage of the total white matter for each case. After measurements had been obtained, each section was further visualised, and a semi‐quantitative rarefaction rating of none, mild, moderate or severe was given, with 10% of sections assessed by another rater giving >95% agreement. An approximate number of vessels affected in the white matter of each case were also recorded.

### Analysis and measurement of angiotensin‐converting enzyme 1 (ACE) protein levels

The measurement of ACE was conducted using western immunoblotting methods that have been previously published [15] and are described in detail in the Supporting Information. Briefly, protein was extracted from 200 mg of frozen middle frontal cortex brain tissue on a subset of 44 cases (22 antihypertensive medicated) used to assess levels of AD proteins [15].

### Statistics

Statistical analyses were carried out using SPSS software (IBM Corp. Released 2016. IBM SPSS Statistics for Macintosh, Version 24.0. Armonk, NY: IBM Corp.). Medication group differences between CVD and ACE measurements were assessed and correlated with AD pathology measurements assessed by Affleck et al [15]. Levene's test of normality was used prior to independent samples *t*‐tests and one‐way analysis of variance (ANOVA), and non‐parametric chi‐square analyses were used to investigate differences or associations in variables of interest between independent groups. Bootstrapping was applied to *t*‐tests and ANOVAs for validation and to calculate robust confidence intervals. Multinomial logistic regression was carried out to determine factors that predicted the severity of SVD covarying for age, sex, post‐mortem delay (PMD), A and B AD neuropathologic scores and hypertensive status. Log‐linear analyses were conducted to assess the association between SVD, CDR, AD neuropathologic change and antihypertensive medication use. Probability (*p*) values <0.05 were judged as statistically significant.

## RESULTS

### Demographic, diagnostic and clinical features

As previously reported [15], the overall age at death was 88 years, with those medicated for hypertension being significantly older, 89 years, compared to those not medicated, 86 years (see table 1; *p* = 0.030). There were no differences in PMD or hypertension duration when comparing between those who were medicated for hypertension or not. No association was detected between sex and medication or hypertensive group. Antihypertensive medication use had a significant association with CDR (see Table 1; *p =* 0.001), revealing that the odds of cases being either a CDR 0 or 0.5 were 3.3 times higher if they were medicated for hypertension than if not. There were no associations detected using chi‐square testing between *ApoE ε4* carrier status and antihypertensive medication use, hypertension, presence of infarct/s or SVD severity. Therefore, *ApoE* genotype was not analysed any further.

### Medications used and CVD

Of the 149 cases, 77 had at least some form of non‐lobar infarction present. There were no differences in the age, PMD, hypertensive status or antihypertensive medication usage between those that had an infarct(s) and those that did not. Fewer women (*p* < 0.001) and cases that had an intermediate/high level of AD change (*p* = 0.009) had non‐lobar infarcts (see Table 2).

**FIGURE 1:**
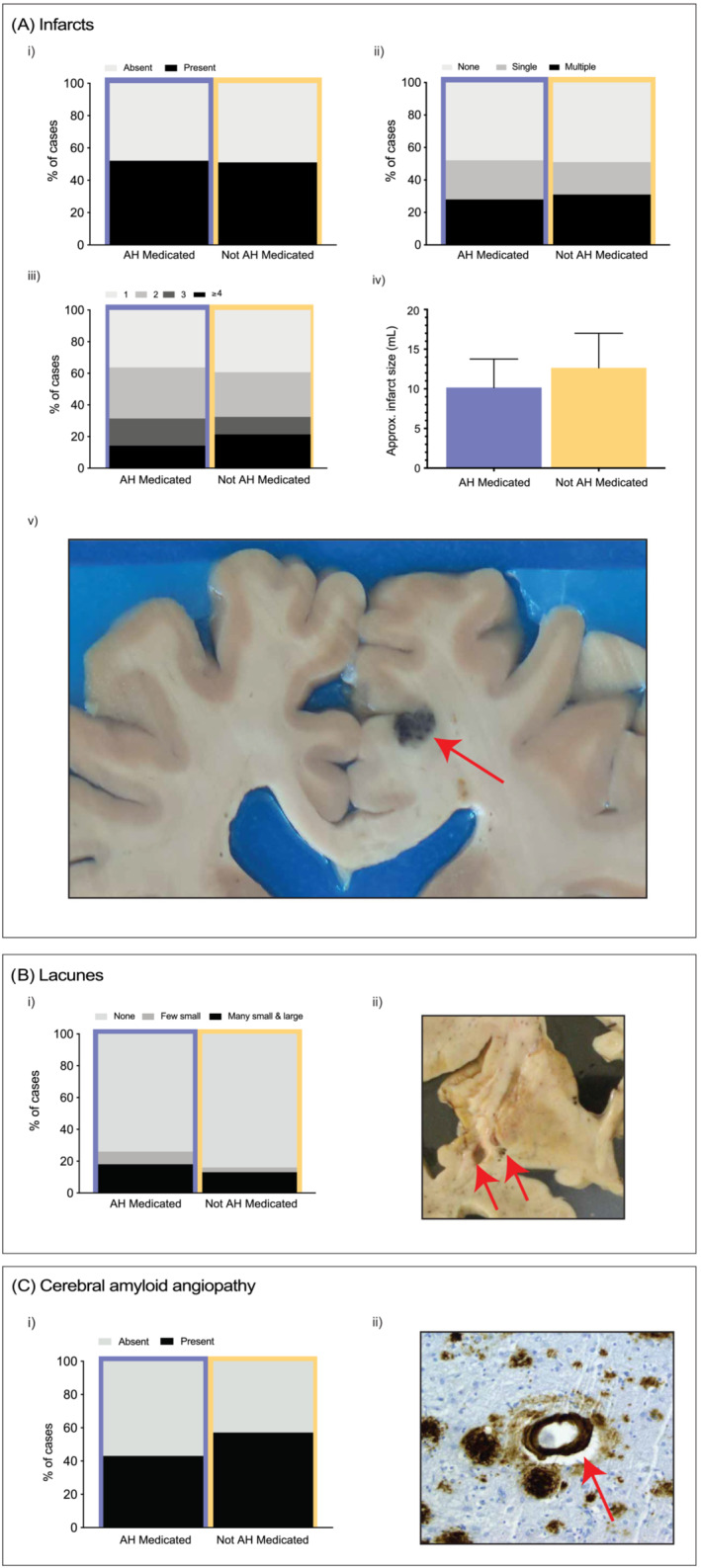
Antihypertensive medication use and cerebrovascular disease pathologies. Stacked bar charts representing the percentage distributions of various cerebrovascular disease pathologies, namely, infarction (A), lacunes (B) and cerebral amyloid angiopathy (C) comparing between antihypertensive medication use (medicated = orchid colour, not medicated = cantaloupe colour). (Ai) Presence or absence of infarct(s). (Aii) Infarct category; presence of none, single or multiple infarcts. (Aiii) Of those that had an infarct(s), proportions of the number of brain regions involved. (Aiv) Of those that had an infarct(s), approximate average volume of infarct(s) was measured in millilitres. Error bars = 95% confidence interval. (Av) Representative macroscopic image of a portion of a brain slice depicting a haemorrhagic infarct (red arrow) seen in the superior frontal gyrus. (Bi) Absence or presence (few small vs many small and large) of lacunes. (Bii) Representative macroscopic image of a portion of a brain slice illustrating lacunes (red arrows) in white matter underlying the insular cortex. (Ci) Presence or absence of cerebral amyloid angiopathy. (Cii) Representative micrograph depicting a blood vessel with cerebral amyloid angiopathy (red arrow).

When assessing the association of antihypertensive medication use with infarcts (Figure 1Av), no association was found between medication usage and the presence or absence of infarcts (*p* = 0.954; see Figure 1Ai) or infarct category (none, single or multiple; *p* = 0.803, Figure 1Aii). There was no association between other vascular‐acting agents taken and the presence or absence of infarcts (Table S2). Of the cases that had infarct(s) present, there was no association seen between antihypertensive medication use and the number of affected infarcted brain regions (*p* = 0.751; Figure 1Aiii). There was also no difference in the approximated infarction size between those medicated or not (*p* = 0.381; Figure 1Aiv and Table S2), except for the few on hypoglycaemic medications which had larger infarcts (Table S2; *p* = 0.049). There were no associations (*p* = 0.279; Figure 1Bi) seen when assessing medication usage and lacunar infarctions (Figure 1Bii and Table S2). There was a decrease in the proportion of antihypertensive medicated cases with CAA (Figure 1Cii); however, this association was not statistically significant (*p* = 0.086; Figure 1Ci) and was not observed with other vascular‐acting agents (Table S2).

**FIGURE 2:**
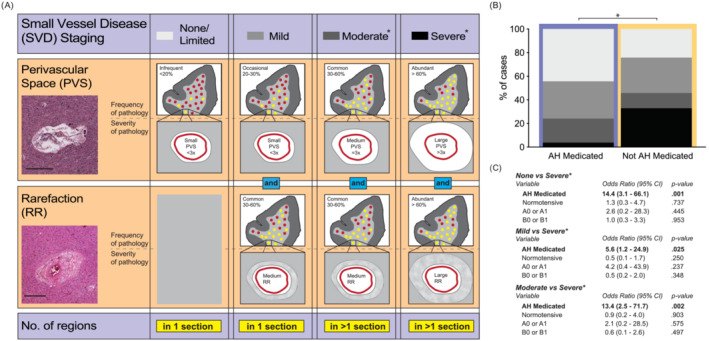
Antihypertensive medication use and small vessel disease severity. (A) Schematic illustrating the neuropathological characteristics and staging of small vessel disease (SVD) used and operationalised in this study from the staging scheme described by Esiri et al [10]. None/limited SVD includes infrequent small perivascular spaces (PVS) in at least one cortical region. Mild SVD includes occasional small PVS and common medium‐sized rarefaction (RR) in at least one cortical region. Moderate SVD includes common medium‐sized PVS and common medium‐sized RR. Severe SVD includes abundant large‐sized PVS and abundant large‐sized RR. Frequency of pathology was assessed by the percentage of vessels that were affected within a sampled region, <20% of vessels affected were rated as infrequent, 20%–30% as occasional, 30%–60% as common and >60% of vessels affected as abundant. The severity of pathology was judged by the size of the lesion (PVS or RR) compared to the thickness of the vessel wall, with a lesion size <3x the thickness of the associated vessel wall rated as small, ≈3x as medium and >3x the vessel wall thickness as large. Large‐rated examples of each type of pathology are shown (inset). Scale bar = 200 μm. *indicates other related pathologies such as spongiosis, cellular infiltrate, congophilic angiopathy and abnormal vessel walls that may or may not be present. (B) Stacked bar charts representing the percentage distribution of the severity of SVD as either none, mild, moderate or severe as outlined in Figure 2A. (C) Pertinent results of the multinomial logistic regression carried out to assess the effect of variables of interest on the likelihood of SVD severity membership using severe SVD as the reference group. For full details of these results, see Table S3.

The only significant result detected in relation to antihypertensive medication use on CVD pathology was with white matter SVD where it was found that if a person was medicated for hypertension, then the overall (not covaried or modelled) odds of them having no or mild SVD was 2.7 times higher than those not medicated (*p* = 0.005; Figure 2B). There was a similar decline in SVD with anti‐angina medication use, but this did not reach statistical significance (*p* = 0.090; Table S2).

Approximately a third of the total cases had either no white matter SVD (*n* = 52) or mild SVD (*n* = 46) with the remaining third roughly split equally between the other white matter SVD severity categories: moderate (*n* = 25) and severe (*n* = 26; see Table 3). No differences were detected between the groups with respect to age, PMD or hypertension duration. There was a significant association between white matter SVD severity and antihypertensive medication use (*p* < 0.001), a result that was duplicated with hypertensive status (*p* = 0.026), likely due to the majority of cases who had hypertension also being medicated for it (see Table 3).

Multinomial logistic regression was carried out to determine the extent antihypertensive medication use related to white matter SVD severity while also factoring in hypertensive status, AD neuropathologic change scores, sex, age at death and PMD. The resulting analysis demonstrated that antihypertensive medication use was a significant predictor of white matter SVD severity. Based on the odds ratio, the likelihood of an individual having either none, mild or moderate white matter SVD severity categories compared with the severe category were 14.4, 5.6 and 13.4 times higher (respectively) if they were medicated for hypertension than if not (see Figure 2C and Table S3).

### Quantitation of regional SVD pathologies in superior frontal lobe white matter

**FIGURE 3:**
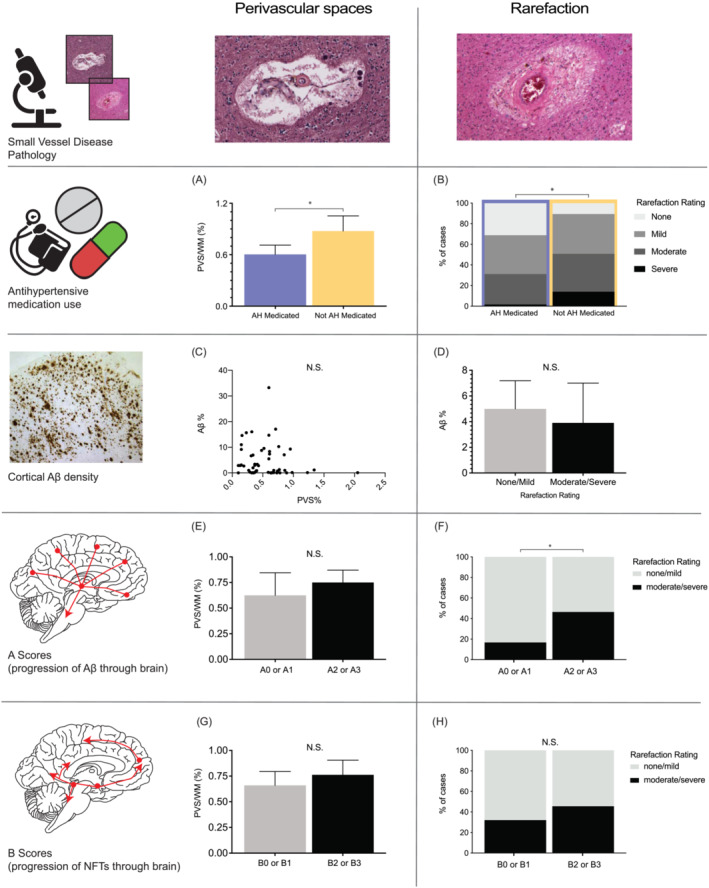
Investigation of the main components of small vessel disease pathology (perivascular space and rarefaction) with antihypertensive medication use, cortical Aβ density, A and B scores for AD neuropathologic change (progression of Aβ and neuritic pathologies through the brain respectively). (A) Column graph depicting percentage area of perivascular spaces averaged across antihypertensive medication groups (medicated = orchid colour, not medicated = cantaloupe colour). Error bars = 95% confidence interval. (B) Stacked bar chart comparing the percentage distributions of rarefaction severity ratings (none, mild, moderate and severe) across antihypertensive medication groups (medicated = orchid colour, not medicated = cantaloupe colour). (C) Scatter plot illustrating the percentage area of cortical Aβ plaques density and perivascular space. (D) Column graph depicting percentage area of cortical Aβ plaques density averaged across rarefaction severity ratings (none, mild, moderate and severe). Error bars = 95% confidence interval. (E) Column graph depicting percentage area of perivascular spaces averaged across the dichotomised A component (A0 or A1 vs A2 or A3) from the ABC score for AD neuropathologic change paradigm. Error bars = 95% confidence interval. (F) Stacked bar chart comparing the percentage distributions of rarefaction severity ratings (none, mild, moderate and severe) across the dichotomised A component (A0 or A1 vs A2 or A3) from the ABC score for AD neuropathologic change paradigm. (G) Column graph depicting percentage area of perivascular spaces averaged across the dichotomised B component (B0 or B1 vs B2 or B3) from the ABC score for AD neuropathologic change paradigm. Error bars = 95% confidence interval. (H) Stacked bar chart comparing the percentage distributions of rarefaction severity ratings (none, mild, moderate and severe) across the dichotomised B component (B0 or B1 vs B2 or B3) from the ABC score for AD neuropathologic change paradigm. Correlation coefficients between severity scores are given in Table S4.

To quantify white matter SVD pathology further, we assessed PVS and RR, the two main pathologies that contribute most to the white matter SVD severity score, in the superior frontal lobe white matter in a subset of cases matched for age at death and AD neuropathologic change (Table 4). White matter PVS were significantly smaller in antihypertensive medicated compared to non‐medicated individuals (Figure 3A). Further, a significant association was detected between the severity of RR and antihypertensive medication use (Figure 3B), revealing that those who were medicated were 2.3 times more likely to have no or mild white matter RR compared to those not medicated.

**TABLE 4:** Case demographics and clinical data—PVS cohort.

Group (*n*)	AH medicated			Hypertensive			AD neuropathologic change		
	Yes (61)	No (57)	Statistics	Yes (59)	No (59)	Statistics	Not/Low (48)	Intermediate/High (70)	Statistics^c^
Age at death, mean years (SD)	89 (5.8)	87 (5.7)	*t*(116) = 1.82 *p* = 0.071 CI [−0.15, 3.9]	88 (5.9)	87 (5.8)	*t*(116) = 0.29 *p* = 0.288 CI [−0.98, 3.22]	89 (6.2)	87 (5.6)	*t*(116) = 1.03 *p* = 0.306 CI [−1.1, 3.2]
Female, *n* (%)	37 (61%)	33 (58%)	𝜒^2^(1) = 0.093 *p* = 0.760	37 (63%)	33 (56%)	𝜒^2^(1) = 0.562 *p* = 0.453	**22** **(46%)**	**48** **(69%)**	**𝜒** ^**2**^ **(1) = 6.10** ***p* = 0.014**
CDR 0 or 0.5, *n* (%)	**31** **(51%)**	**15** **(26%)**	**𝜒** ^**2**^ **(1) = 7.44** ***p* =** 0**.006**	24 (41%)	22 (37%)	𝜒^2^(1) = 0.143 *p* = 0.706	**29** **(60%)**	**17** **(24%)**	**𝜒** ^**2**^ **(1) = 15.63** ***p* < 0.001**
PMD, mean hours (SD)	19 (16.6)	23 (17.4)	*t*(116) = −1.16 *p* = 0.249 CI [−9.3, 2.4]	21 (15.7)	21 (18.4)	*t*(116) = −0.17 *p* = 0.858, CI [−6.2, 5.5]	**25** **(19.5)**	**18** **(14.5)**	***t*(116) = 2.41** ***p* = 0.018** **CI [1.4, 13.7]**
Hypertensive, *n* (%)	**44** **(72%)**	**15** **(26%)**	**𝜒** ^**2**^ **(1) = 24.7** ***p* < 0.001**	—	—	—	23 (48%)	36 (51%)	𝜒^2^(1) = 0.140 *p* = 0.708
Hypertension duration, mean years (SD) [*n*]^a^	14 (9.8) [41]	13 (11.5) [15]	*t*(54) = 0.27 *p* = 0.791 CI [−6.7, 6.8]	14 (10.2) [56]	—	—	15 (9.6) [21]	13 (10.6) [35]	*t*(54) = 0.73 *p* = 0.466 CI [−2.8, 6.9]
VCING criteria									
Low, *n* (%)	32 (53%)	25 (44%)	𝜒^2^(2) = 1.67 *p* = 0.435	34 (58%)	23 (39%)	𝜒^2^(2) = 0.4.24 *p* = 0.120	21 (44%)	36 (51%)	𝜒^2^(2) = 3.59 *p* = 0.166
Moderate, *n* (%)	16 (26%)	14 (25%)		13 (22%)	17 (29%)		10 (21%)	20 (29%)	
High, *n* (%)	13 (21%)	18 (32%)		12 (20%)	19 (32%)		17 (35%)	14 (20%)	
Apolipoprotein E^b^ (*N* = 80), *n* (%)	46 (58%)	34 (43%)	N/A	45 (56%)	35 (44%)	N/A	29 (36%)	51 (64%)	N/A
Apolipoprotein E *ε4* carrier, *n* (% of column metric)	13 (28%)	16 (47%)	𝜒^2^(1) = 3.0 *p* = 0.084	15 (33%)	14 (40%)	𝜒^2^(1) = 0.379 *p* = 0.538	**3** **(10%)**	**26** **(51%)**	**𝜒** ^**2**^ **(1) = 13.21** ***p* < 0.001**

*Notes*: Hypertensive cases based on clinical assessment. Statistically significant results (*p* < 0.05) are in bold.

Abbreviations: N, number of cases, PMD, post‐mortem delay; SD, standard deviation.

^a^ Average duration in years of hypertensive cases. Three cases did not have hypertension duration data due to incomplete clinical records (i.e. did have confirmation of hypertensive status but no information on the date of diagnosis).

^b^ ApoE data not available on the entire cohort, was available on 94 cases. ^c^Independent samples *t*‐tests were used for quantitative variables with the addition of bootstrapping to validate and calculate robust confidence intervals, chi‐square tests were used for qualitative variables.

^c^ Independent samples *t*‐tests were used for quantitative variables with the addition of bootstrapping to validate and calculate robust confidence intervals, chi‐square tests were used for qualitative variables.

### Angiotensin‐converting enzyme (ACE) levels and regional SVD

**FIGURE 4:**
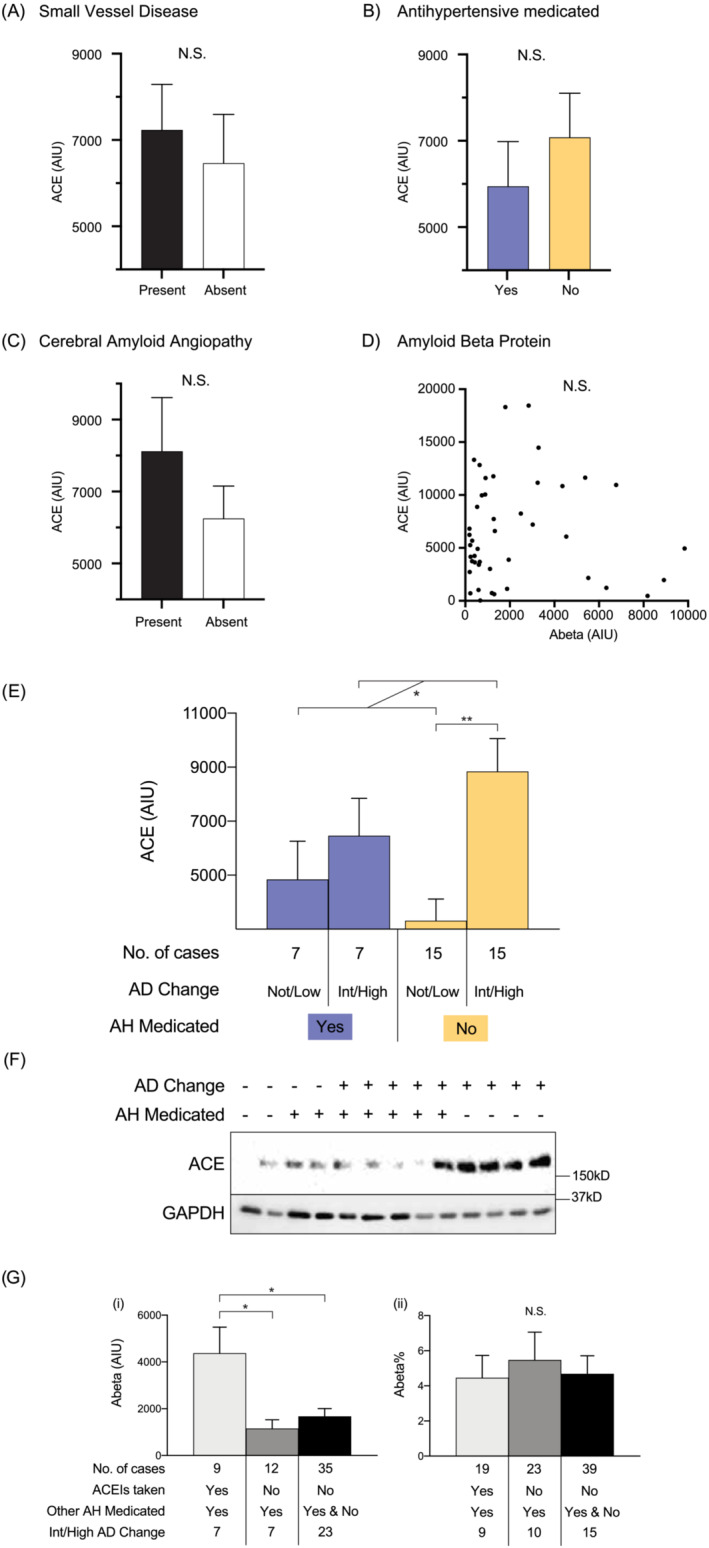
Associations between ACE levels, pathologies and antihypertensive medication use. (A) Bar graph showing no difference in frontal cortex ACE levels and the presence or absence of pathological small vessel disease. (B) Bar graph showing no difference in frontal cortex ACE levels in those medicated with antihypertensive medications during life or not. (C) Bar graph showing no difference in frontal cortex ACE levels in those with or without cerebral amyloid angiopathy. (D) Scatter plot illustrating frontal ACE levels vs Aβ levels. (E) Bar graph showing levels of ACE detected in the frontal cortex of cases who were (orchid colour) and were not (cantaloupe colour) medicated with antihypertensive medications during life further dichotomised into a not/low or intermediate/high level of AD neuropathologic change as measured in arbitrary intensity units (AIU) from western immunoblotting. Case types and number of cases are illustrated in key underneath bar graph. (F) Representative western immunoblots of the data presented in (A). Lanes denoted as AD change “−” indicate not/low level of AD change, while those denoted as “+” indicate an intermediate/high level of AD change. Lanes denoted as AH medicated “−” indicated not antihypertensive medicated, while those denoted as AH medicated “+” indicate antihypertensive medicated cases. (G) Bar graphs illustrating levels of Aβ detected in the frontal cortex via western immunoblotting (i) and percentage of Aβ plaque area as measured via immunohistochemistry (ii) in those who had and had not taken ACEIs. Cases types and number of cases are illustrated in key underneath bar graph. Error bars = standard error of the mean. *p < 0.05 and **p < 0.01.

ACEI are a class of antihypertensive medications that act on ACE activity, and ACE activity has been shown to be increased in association with Aβ load, colocalising perivascularly with CAA [32], while ACE protein levels are changed in AD [33, 34, 35]. Analysis of ACE levels in the present study revealed no difference in the regional levels of ACE between cases with and without SVD (Figure 4A) or in those taking or not taking antihypertensive medications (Figure 4B). In the groups with or without SVD, there was a relatively equal proportion of cases that were ACEI users (24% and 21%, respectively), indicating an equivalent impact of ACEI on these measurements. In addition, there was no difference or relationship detected with white matter PVS and rarefaction (data not shown), nor did the overall multinomial logistic regression model (Figure 2C) change when ACEI users were analysed by themselves or taken out (data not shown). As ACEI are thought to impact Aβ deposition [36], we further assessed ACE levels and CAA, and ACE levels vs Aβ levels. There was no difference in the regional levels of ACE between cases with and without CAA (Figure 4C) and no correlation between regional ACE and Aβ levels (Figure 4D).

### Associations between CVD, ACE brain tissue levels and AD pathologies

In the superior frontal lobe, there was no correlation between the amount of PVS in the white matter and the amount of Aβ plaques detected in the overlying grey matter (Figure 3C). In addition, the amount of Aβ plaques did not differ significantly by the severity of white matter RR (Figure 3D). ACE levels were higher in cases with AD neuropathic change and were not associated with overall antihypertensive medication use (Figure 4E,F). Assessment of ACEI use revealed that this could be due to a greater proportion of AD cases in the ACEI user group (78%) compared to the non‐ACEI user group (58%). While the amount of cortical Aβ plaques was not affected by ACEI use (Figure 4Gii), there was an increase in cortical Aβ protein levels in antihypertensive medication users who had taken ACEIs compared to non‐ACEI users (Figure 4Gi). This suggests there was a higher Aβ level/plaque in the ACEI with AD group.

When assessing the progression of plaque (A score) and NFTs (B score) through the brain, there were no differences in white matter PVS size and the progression of these AD pathologies (Figure 3E,G), but there was a greater proportion of cases with moderate/severe white matter RR rating that had higher A scores (Figure 3F). Those with none or mild white matter RR were 4.3 times more likely to have an A0 or A1 Aβ plaque score compared to those with moderate or severe RR. White matter RR did not have a significant association, however, with their B scores (Figure 3H).

### Clinical associations

Considering that white matter SVD severity was the only CVD neuropathological metric to significantly associate with antihypertensive medication use, and that being medicated for hypertension resulted in a significantly greater likelihood of less white matter SVD, the next set of analyses aimed at examining the relationship between antihypertensive medication use, white matter SVD severity and the clinical and neuropathological correlates of AD. Three‐way chi‐square analyses revealed that antihypertensive medication use was significantly associated with reduced CDR (Figure S3A) and A scores (Figure S3B) but only at the moderate and severe levels of white matter SVD. No associations were detected when conducting the same analyses with B score (Figure S3C) and AD neuropathologic change (Figure S3D).

## DISCUSSION

Antihypertensive medications are routinely used to decrease the risk of both cardiovascular and cerebrovascular diseases, with neuroimaging evidence that such medications protect against white matter damage [17, 18, 19, 20, 21]. While SVD was originally defined pathologically, it is now primarily defined by neuroimaging characteristics as either CAA or deep perforating arteriopathy [37, 38], and it is the deep white matter damage that appears to benefit most from antihypertensive treatment (see above). Our study is the first histopathological study to confirm that antihypertensive medication use is associated with less white matter SVD and not with other CVD pathologies, with treatment particularly reducing PVS and RR. Those medicated were 2.7 times more likely to have no or mild white matter SVD, with antihypertensive treatment use as a significant predictor of SVD severity. While these data are in keeping with neuroimaging data showing a reduction in the progression of white matter hyperintensities following antihypertensive medication use [38], we provide more evidence of the cellular changes associated with this effect.

When quantifying the severity of SVD pathologies in frontal white matter, we found a significant decrease in PVS in those on antihypertensive medications. In neuroimaging studies, white matter PVS has been associated with hypertension and cerebral SVD [39, 40, 41], with increasing quantities of white matter PVS related to the severity of white matter hyperintensities [39, 42]. The association between white matter hyperintensities and enlarged PVS has been recently replicated in rats [41, 43, 44] with distinct proteomic brain changes compared with CAA [44]. While the quantitation of PVS is becoming more common in neuroimaging studies [41, 45], it is not a core pathology in the rubric used to assess vascular pathologies in the Vascular Cognitive Impairment Neuropathologic Guidelines (VCING), which include large infarcts, moderate to severe occipital leptomeningeal CAA and moderate to severe occipital white matter arteriosclerosis rather than white matter PVS [7]. These guidelines were designed to capture pathologies associated with cognitive impairment, with evidence that increasing enlargement of white matter PVS is a marker for increased risk of cognitive decline and dementia [46]. In pathological cohorts of AD ± CVD compared with controls without CVD, there is an increase in enlarged PVS in AD cases [47]. Our data importantly show that these white matter PVS are reduced with antihypertensive medication use.

In the present study, we also found less severe white matter RR in those on antihypertensive medications. Histopathological RR is a measure of overall white matter integrity and is defined by the extent of tissue attenuation or vacuolisation around small blood vessels, the density of reactive astrocytes and eventually the degree of myelin loss [48]. These changes are consistent with increased permeability of the blood–brain barrier and tissue oedema that associate with hypertension [49]. One of the known drivers of fluid movement in the perivascular space around small vessels is vascular pulsations with blood pressure elevation reducing the net flow of perivascular fluid [41]. Increased intracranial pulsatility is associated with a greater volume of white matter hyperintensities [41], a measure that can be reduced with antihypertensive medications (see above). In brain donors exposed to head injury, the severity of histopathological RR correlates with the total volume of white matter hyperintensities [41, 50], confirming this association at a tissue level. Our quantitative data suggest that SVD‐associated white matter RR can be ameliorated by the use of antihypertensive medications.

Our analysis also found that in those with moderate to high white matter SVD severity, antihypertensive medication use was associated with lower CDR and A scores (indicating less pathological progression of Aβ through the brain). In a case–control study of AD using the National Alzheimer's Coordinating Center dataset [9], white matter RR was significantly associated with faster progression of clinical AD dementia, but not independently of AD pathologies. Our data using a broader inclusion of non‐lobar vascular pathologies suggest that the association of white matter RR and progression of Aβ through the brain occurs in those with moderate to severe SVD independently of AD pathologies. This suggests that white matter RR in those with SVD can still be ameliorated by the use of antihypertensive medication use and reduce associated cognitive impairment.

SVD occurs in 30% of those with ischaemic stroke, 80% of those with intracerebral haemorrhage, and 45% of those with dementia [38]. In our study, antihypertensive medication use did not associate with infarction (presence, type, number and size) or lacunes, although those with lobar infarcts were excluded. In one histological study assessing the impact of angiotensin receptor blockers [51], hypertensive participants receiving these medications had more frequent large vessel infarcts and haemorrhages and were more likely to have had large stroke/s. As these pathologies were excluded in the present study, it is likely that antihypertensive treatment in those with very severe CVD may differ from the effects identified in the present study of those with less dramatic CVD.

Considering that ACE has been shown to be an Aβ‐degrading enzyme [52] and associates with CAA Aβ [32] and increasing B score [34] and that ACEIs are risk factors for impaired human cognition [36], we assessed cortical ACE levels and pathologies in association with white matter SVD and ACEI users. We found no difference in the regional levels of ACE between cases with and without white matter SVD or in those taking or not taking antihypertensive medications. These groups had only 21%–24% of cases taking ACEI, potentially impacting this result, although further multinominal logistic regressions suggested that this was not the case. While cortical ACE protein levels have been reported to be slightly reduced in AD in one study [33], our study is consistent with others [32, 34] that have found cortical ACE levels are higher in cases with AD neuropathic change. While this could suggest a reactive change to the increase in cortical Aβ in the AD brain [32, 34], we believe that in our study (and potentially others reporting higher cortical ACE levels), this was due to a greater proportion of AD cases using ACEI (78%) compared to the non‐AD group (58%). Importantly, there was no correlation between regional ACE and Aβ levels in general, and the amount of cortical Aβ plaques overall was not affected by ACEI use. There was also no difference in cortical ACE levels in those with or without CAA. However, there was an increase in cortical Aβ protein levels in ACEIs compared to non‐ACEI users. Due to the higher numbers of cases with AD neuropathologic change who were ACEI users, this suggests there was a higher Aβ level/plaque in these cases. Overall, the data are consistent with ACEI use increasing the concentration of Aβ in plaques in those with AD neuropathologic change despite these cases having higher cortical ACE protein levels.

Our study has limitations in that it mainly assessed the frontal lobe and we excluded lobar infarctions. The exclusion of lobar infarctions was for practical reasons but means that more advanced disease was not assessed. Thus, our data can be considered to represent earlier stages of CVD overall. The frontal lobe was chosen as CVD is found in this cortical region more often than others and it is an anatomical region commonly used in histopathological studies of CVD [53]. However, a large meta‐analysis shows that all antihypertensive medication regimens achieve similar blood pressure control [54]. We only assessed cases over the age of 75 years as CVD is a significant cause of death in Australia in this age population (see https://www.aihw.gov.au/reports/life-expectancy-death/deaths-in-australia/contents/leading-causes-of-death). Different associations may occur in those at younger ages. Lastly, the SVD severity data need to be replicated in larger groups, particularly in the antihypertensive medication group where there was a strong association with less severe SVD and few cases with severe SVD that were taking antihypertensive medications.

Our study also has considerable strengths. It is the first histopathological study to assess the association between antihypertensive medication use and different types of CVD. We also used both semiquantitative and quantitative measures to assess the main SVD pathologies associated with antihypertensive medication use and included AD neuropathologies in the statistical analyses. Our results provide strong evidence of significantly decreased white matter SVD and better cognition with antihypertensive medication use, confirming results from many studies using neuroimaging techniques. We provide more detailed evidence of the tissue differences with antihypertensive medication use than the data from neuroimaging studies and show that only reduced white matter SVD severity and not other CVD pathologies are ameliorated by antihypertensive medication use. When investigating the different aspects of pathological SVD, both PVS and RR were the main white matter pathologies reduced by antihypertensive medication use, but only the reduction in white matter RR severity was associated with a less extensive spread of Aβ through the brain. This suggests that tissue oedema around small blood vessels in the white matter is important for trafficking Aβ through the brain in those with SVD, while additional Aβ trafficking mechanisms may also occur in cases with AD [55]. SVD is known, like AD, to be progressive, but its progression is not necessarily linear in nature and appears more variable in those with moderate or severe presentations [56]. Our data suggest that antihypertensive medication use mostly reduces white matter SVD, and mainly RR in those with moderate or severe white matter SVD.

## AUTHOR CONTRIBUTIONS

Andrew J. Affleck planned the experiments, extracted and produced all data, analysed the data with Glenda M. Halliday and drafted the manuscript. Perminder S. Sachdev planned the experiments and reviewed all data and the manuscript. Glenda M. Halliday planned the experiments, analysed the data with Andrew J. Affleck and revised the initial manuscript draft.

## CONFLICT OF INTEREST STATEMENT

PSS consulted for Biogen Australia and Roche Australia in 2020 and 2021. The other authors have no conflicts of interest. The Editors of Neuropathology and Applied Neurobiology are committed to peer‐review integrity and upholding the highest standards of review. As such, this article was peer‐reviewed by independent, anonymous expert referees, and the authors (including GH) had no role in either the editorial decision or the handling of the paper.

## ETHICS STATEMENT

All cases were sourced from the human brain collection held at the Sydney Brain Bank for research purposes, as approved by the University of New South Wales Human Research Ethics Committee (approval no. HC200026). All participants and/or their senior available next of kin consented to brain and clinical and research data retrieval for future research in ethically approved studies. Our particular research study was approved by the University of New South Wales Human Research Ethics Committee (approval no. HC15613).

### PEER REVIEW

The peer review history for this article is available at https://www.webofscience.com/api/gateway/wos/peer-review/10.1111/nan.12922.

## Supporting information


**Figure S1.** Case selection flow chart.
**Figure S2.** Venn diagram illustrating the type and number of cases involved in the study and the overlap with variables of interest (AD change, CVD presence and antihypertensive medication use).
**Figure S3.** A) Stacked bar chart comparing the percentage distributions of CDR (0 or 0.5, 1, 2 and 3) across dichotomised SVD severity (none or mild vs. moderate or severe) and antihypertensive medication use groups (medicated = orchid colour, not medicated = cantaloupe colour). B) Stacked bar chart comparing the percentage distributions of the A component (A0, A1, A2 and A3) from the ABC score for AD neuropathologic change paradigm across dichotomised SVD severity (none or mild vs. moderate or severe) and antihypertensive medication use groups (medicated = orchid colour, not medicated = cantaloupe colour). C) Stacked bar chart comparing the percentage distributions of the B component (B0, B1, B2 and B3) from the ABC score for AD neuropathologic change paradigm across dichotomised SVD severity (none or mild vs. moderate or severe) and antihypertensive medication use groups (medicated = orchid colour, not medicated = cantaloupe colour). D) Stacked bar chart comparing the percentage distributions of AD neuropathologic change level (not, low, intermediate and high) across dichotomised SVD severity (none or mild vs. moderate or severe) and antihypertensive medication use groups (medicated = orchid colour, not medicated = cantaloupe colour).
**Table S1.** Other medications.
**Table S2.** Other vascular acting agents taken.
**Table S3.** Multinomial logistic regression statistics – SVD.
**Table S4.** SVD severity and AD severity score corelations.

## Data Availability

We will be making the data available but have not done so yet.
