# Supplementary material for: Past antihypertensive medication use is associated with lower levels of small vessel disease and lower Aβ plaque stage in the brains of older individuals
Source: Neuropathol Appl Neurobiol. 2023 Aug 1;49(4):e12922. doi: 10.1111/nan.12922 (PMC10947144; doi:10.1111/nan.12922)
Supplement: Supplementary file 1 — Figure S1. Case selection flow chart. Figure S2. Venn diagram illustrating the type and number of cases involved in the study and the overlap with variables of interest (AD change, CVD presence and antihypertensive medication use). Figure S3. A) Stacked bar chart comparing the percentage distributions of CDR (0 or 0.5, 1, 2 and 3) across dichotomised SVD severity (none or mild vs. moderate or severe) and antihypertensive medication use groups (medicated = orchid colour, not medicated = cantaloupe colour). B) Stacked bar chart comparing the percentage distributions of the A component (A0, A1, A2 and A3) from the ABC score for AD neuropathologic change paradigm across dichotomised SVD severity (none or mild vs. moderate or severe) and antihypertensive medication use groups (medicated = orchid colour, not medicated = cantaloupe colour). C) Stacked bar chart comparing the percentage distributions of the B component (B0, B1, B2 and B3) from the ABC score for AD neuropathologic change paradigm across dichotomised SVD severity (none or mild vs. moderate or severe) and antihypertensive medication use groups (medicated = orchid colour, not medicated = cantaloupe colour). D) Stacked bar chart comparing the percentage distributions of AD neuropathologic change level (not, low, intermediate and high) across dichotomised SVD severity (none or mild vs. moderate or severe) and antihypertensive medication use groups (medicated = orchid colour, not medicated = cantaloupe colour). Table S1. Other medications. Table S2. Other vascular acting agents taken. Table S3. Multinomial logistic regression statistics – SVD. Table S4. SVD severity and AD severity score corelations. [file NAN-49-0-s001.pdf]

Past antihypertensive medication use is associated with lower levels of small vessel disease and lower Aβ plaque stage in brains of older individuals

Andrew J. Affleck[a,b], Perminder S. Sachdev[b,c], Glenda M. Halliday[a,d,e]

[a]Neuroscience Research Australia (NeuRA), Sydney, Australia

[b]Centre for Health Brain Ageing (CHeBA), Discipline of Psychiatry and Mental Health, Faculty of Medicine, University of New South Wales, Sydney, Australia

[c]Neuropsychiatric Institute, the Prince of Wales Hospital, Sydney, Australia.

[d]School of Medical Sciences, Faculty of Medicine, University of New South Wales, Sydney, Australia

[e]Brain and Mind Centre & Faculty of Medicine and Health School of Medical Sciences, University of Sydney, Sydney, Australia

Abbreviations

ACE – Angiotensin converting enzyme

AH – Antihypertensive

AIU – Arbitrary intensity units

BCA – Bicinchoninic acid

CDR – Clinical dementia rating

CVD – Cerebrovascular disease

ECL – Enhanced chemiluminescence

EDT – Ethylenediaminetetraacetic acid

GAPDH – Glyceraldehyde 3-phosphate dehydrogenase

LDS – Lithium dodecyl sulfate

mg – Milligram

mL – Millilitre

NaCl – Sodium chloride

NaN_3 – Sodium azide

PAGE – Polyacrylamide gel electrophoresis

RPM – Revolutions per minute

SDS – Sodium dodecyl-sulfate

SVD – Small vessel disease

TBS – Tris buffered saline

TBST – Tris buffered saline with Tween20

V – Volts

Analysis and measurement of ACE protein levels

The measurement of ACE was conducted using western immunoblotting methods that have been previously described in detail [1]. Protein was extracted from the 200 mg of frozen middle frontal cortex brain tissue on a subset of 44 cases (22 antihypertensive medicated) used to assess levels of AD proteins [1]. Samples were homogenised (in 20 mM Tris, 150mM NaCl, 5mM EDTA and 0.02% NaN3 with a Roche complete EDTA-free protease inhibitor cocktail tablet added prior to use) then sonicated (Intertek ultrasonic cleaner) before being centrifuged (Beckman Optima L-90K ultracentrifuge at 37,000 RPM for 60 minutes at 4°C). The resulting supernatant was collected as the TBS soluble fraction while the remaining pellet was resuspended and further homogenised, sonicated in 2 mL sodium dodecyl sulfate (SDS) solubilisation buffer, left to incubate at room temperature on a shaking table for 60 minutes before being centrifuged at 37,000 RPM for 30 minutes at 25°C. The resulting supernatant was collected as the SDS soluble fraction which was used to investigate ACE protein levels. Total protein concentrations were determined by bicinchoninic acid (BCA) protein assay kit (Pierce Biotechnology, Rockford, IL) as per the manufacturer's instructions. Thirty micrograms of total protein from the SDS soluble fraction was combined with 1x LDS sample buffer and 2.5% β-mercaptoethanol, heated at 95°C for 5 minutes and then separated by reducing 7.5% SDS-PAGE gels, at a constant 110 V for approximately 90 minutes, before being transferred to nitrocellulose membranes (Bio-Rad, Hercules, CA), at a constant 12 V for 60 minutes, using the Mini Gel Tank and Mini Blot Module (Life Technologies, Carlsbad, CA). Membranes were blocked for 1 hour in 5% skim milk powder in tris-buffered saline with 0.1% (v/v) Tween20 (TBST) after antigen retrieval (microwave incubation in boiling citrate buffer for 2 minutes on each side of the membrane). Membranes were then probed for monoclonal rabbit anti-angiotensin converting enzyme 1 (abcam, Cat no. ab75762 [clone ERP2757], diluted 1:1,000 in 1% skim milk TBST) overnight at 4°C on a rocker, washed in TBST (3 x 10 minutes) the following morning and then probed for goat anti-rabbit horseradish peroxidase secondary antibody (Thermo Scientific, Cat no.

31460, diluted 1:5,000 in 1% skim milk TBST) for 1 to 2 hours rocking at room temperature. Membranes were briefly incubated in Clarity western enhanced chemiluminescence (ECL) substrate (Bio-Rad) and visualised using a Chemidoc MP digital imaging system (Bio-Rad). After image capture, membranes were then re-probed with glyceraldehyde 3-phosphate dehydrogenase (GAPDH) (Sigma, Cat G8795, diluted 1:10,000 in 1% skim TBST, then donkey anti-mouse Alexa Fluor 488 abcam, Cat no. 150105, diluted 1:5,000 in TBST) before being visualised again using the Chemidoc system. Membranes were visualised using the same imaging protocol and exposure times for all membranes that were imaged together. Quantification of protein band intensity was carried out using the gels plugin in Fiji (Image J, National Institutes of Health, Bethesda, MD) being expressed as arbitrary intensity units (AIU) normalised to GAPDH as a protein loading control and standardised to an internal control so that comparison could be made between membranes. All western blots were replicated with resulting AIUs averaged. Rare data points that exceeded two standard deviations from the mean were considered extreme outliers and subsequently removed from the analysis.

Reference

1 Affleck AJ, Sachdev PS, Stevens J, Halliday GM. Antihypertensive medications ameliorate Alzheimer's disease pathology by slowing its propagation. Alzheimers Dement (N Y) 2020; 6: e12060

Supplementary Figure 1. Case selection flow chart

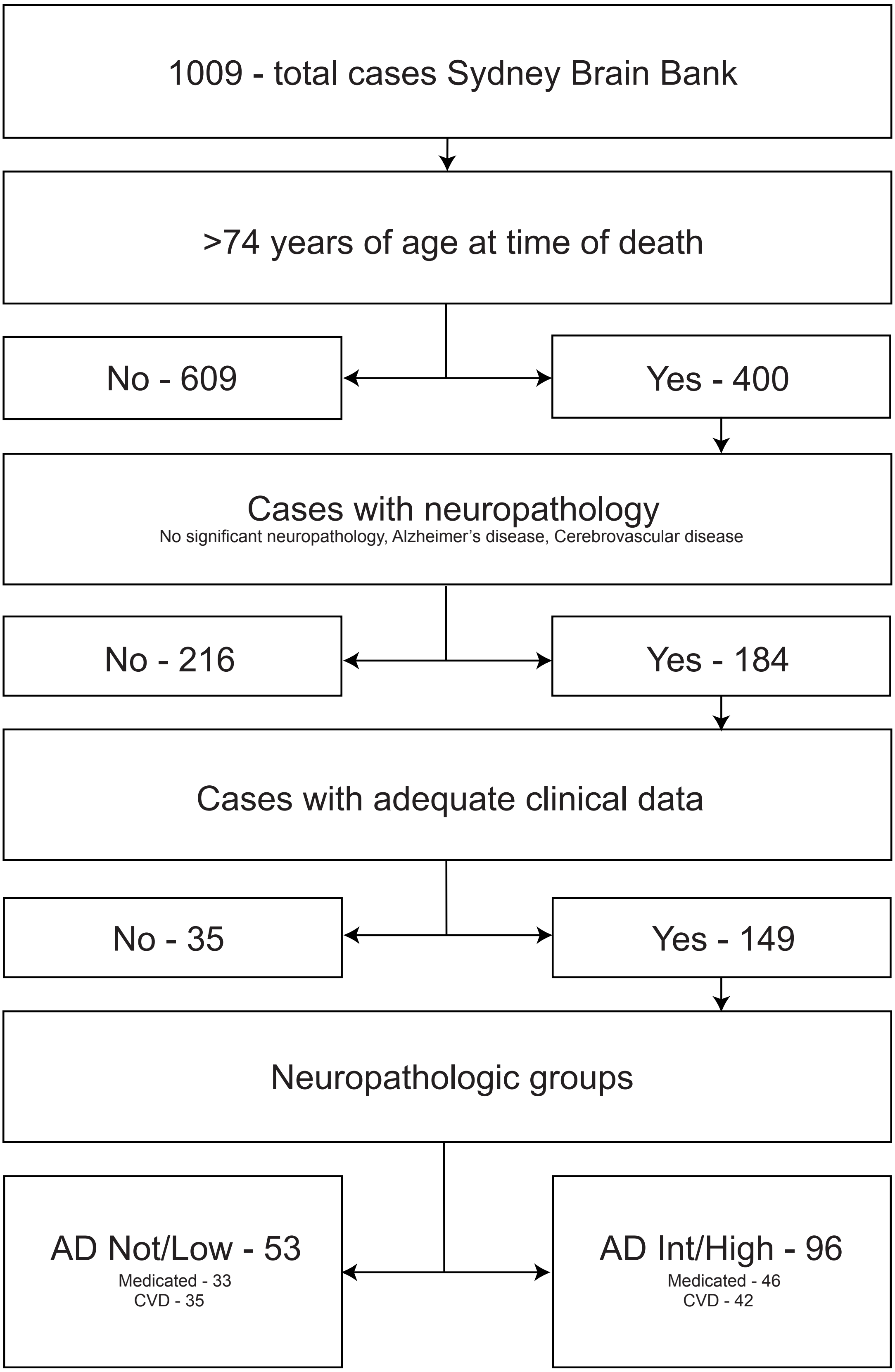

Supplementary Figure 2. Venn diagram illustrating the type and number of cases involved in the study and the overlap with variables of interest (AD change, CVD presence and antihypertensive medication use)

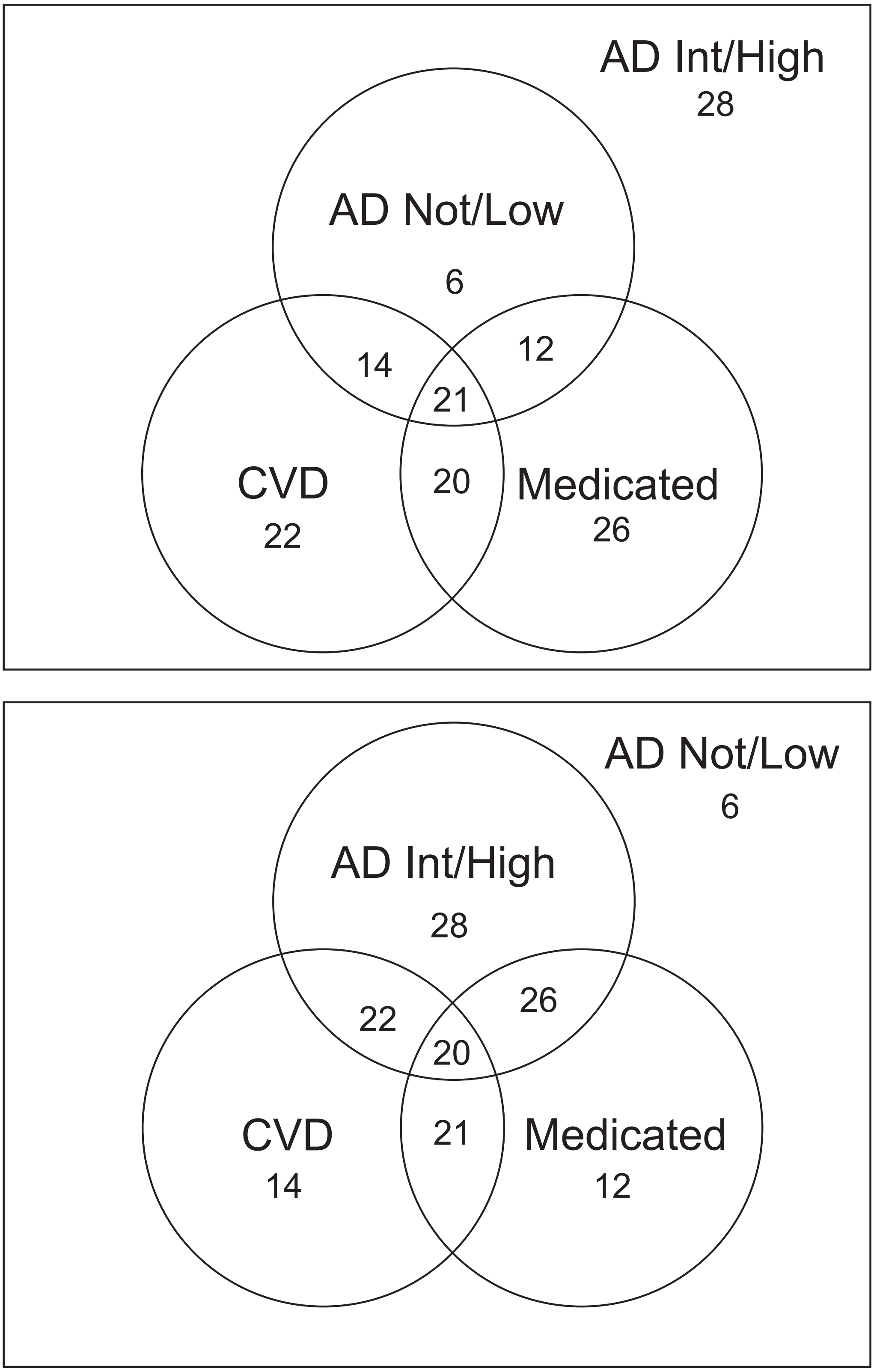

Supplementary Figure 3. A) Stacked bar chart comparing the percentage distributions of CDR (0 or 0.5, 1, 2 and 3) across dichotomised SVD severity (none or mild vs. moderate or severe) and antihypertensive medication use groups (medicated = orchid colour, not medicated = cantaloupe colour). B) Stacked bar chart comparing the percentage distributions of the A component (A0, A1, A2 and A3) from the ABC score for AD neuropathologic change paradigm across dichotomised SVD severity (none or mild vs. moderate or severe) and antihypertensive medication use groups (medicated = orchid colour, not medicated = cantaloupe colour). C) Stacked bar chart comparing the percentage distributions of the B component (B0, B1, B2 and B3) from the ABC score for AD neuropathologic change paradigm across dichotomised SVD severity (none or mild vs. moderate or severe) and antihypertensive medication use groups (medicated = orchid colour, not medicated = cantaloupe colour). D) Stacked bar chart comparing the percentage distributions of AD neuropathologic change level (not, low, intermediate and high) across dichotomised SVD severity (none or mild vs. moderate or severe) and antihypertensive medication use groups (medicated = orchid colour, not medicated = cantaloupe colour).

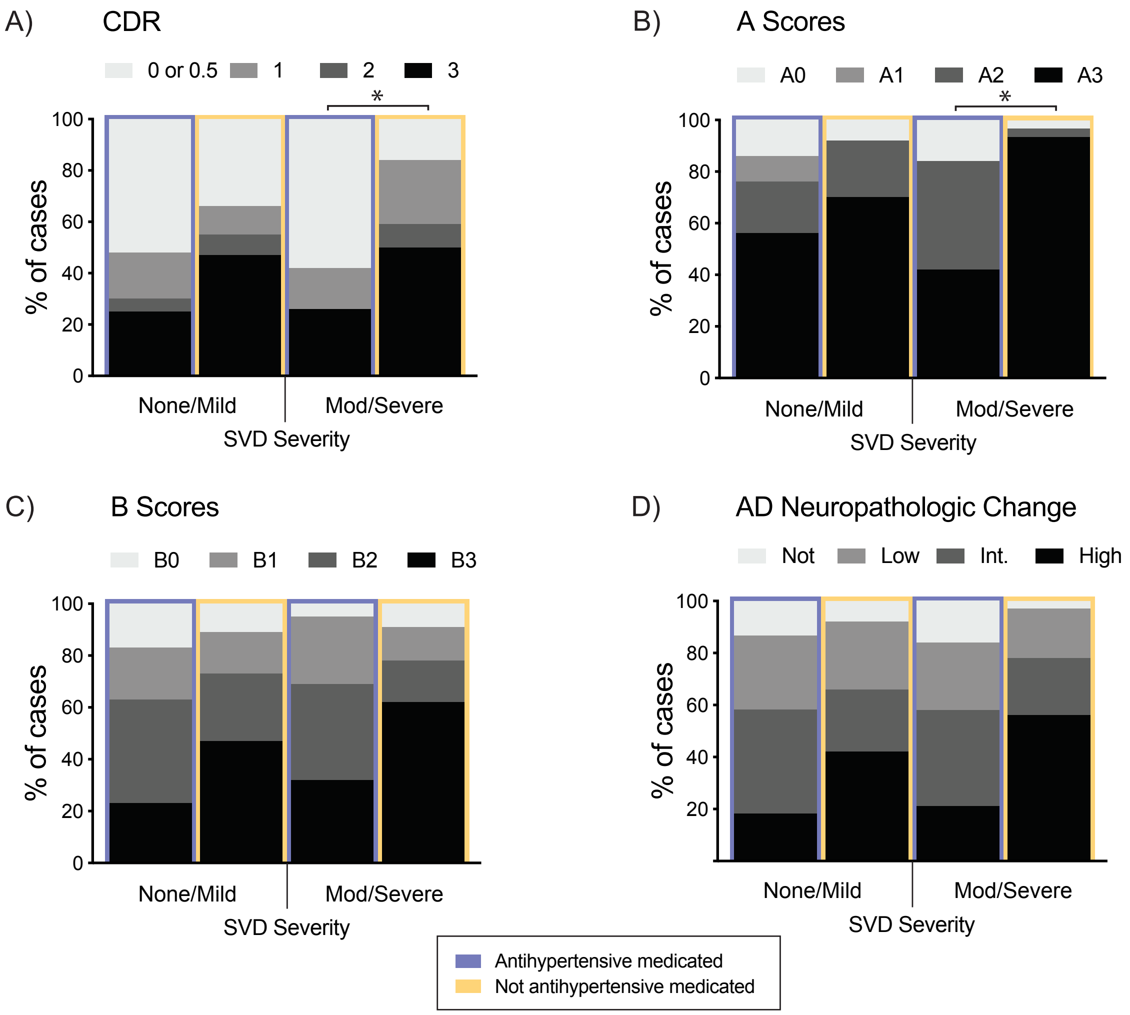

Supplementary Table 1. Other medications

Other Medications taken	Antihypertensive Medicated		
	Yes (79)	No (70)	Total (149)
Aspirin, n (%)	42 (53%)	10 (14%)	52 (35%)
Multi-vitamins & Minerals, n (%)	41 (52%)	17 (24%)	58 (39%)
Simple analgesics & antipyretic (not including aspirin), n (%)	24 (30%)	13 (19%)	37 (25%)
Hyperacidity and related medications, n (%)	22 (28%)	8 (11%)	30 (20%)
Anti-angina medications, n (%)	22 (28%)	2 (3%)	24 (16%)
Hypolipidemic medications, n (%)	21 (27%)	0	21 (14%)
Laxatives, n (%)	20 (25%)	14 (20%)	34 (23%)
Anti-Inflammatory, n (%)	18 (23%)	5 (7%)	23 (15%)
Anti-depressants, n (%)	17 (22%)	9 (13%)	26 (17%)
Cardiac inotropic medications, n (%)	15 (19%)	2 (3%)	17 (11%)
Anti-psychotics, n (%)	14 (18%)	17 (24%)	31 (21%)
Sedatives/Hypnotics, n (%)	14 (18%)	12 (17%)	26 (17%)
Anti-coagulants (not including aspirin), n (%)	14 (18%)	3 (4%)	17 (11%)
Bronchodilators, n (%)	13 (17%)	3 (4%)	16 (11%)
Calcium/bone medications	13 (17%)	1 (1%)	14 (9%)
Anti-anxiety medications, n (%)	12 (15%)	6 (9%)	18 (12%)
Topical corticosteroids, n (%)	7 (9%)	4 (6%)	11 (7%)
Anticonvulsants, n (%)	6 (8%)	6 (9%)	12 (8%)
Acetylcholinesterase inhibitor, n (%)	5 (6%)	7 (10%)	12 (8%)
Hypoglycaemic medications, n (%)	5 (6%)	1 (1%)	6 (4%)
Antiarrhythmic agents, n (%)	4 (5%)	1 (1%)	5 (3%)

Supplementary Table 2. Other vascular acting agents taken

	Anti-angina (24)	Hypo-lipidemic (21)	Cardiac inotropic (17)	Hypo-glycaemic (6)	Anti-arrhythmia (5)
Infarct, n (%)<br>Present (77)<br>Absent (72)	 15 (20%)<br>9 (13%)	 11 (14%)<br>10 (14%)	 11 (14%)<br>6 (8%)	 4 (5%)<br>2 (3%)	 3 (4%)<br>2 (3%)
Statistics	χ^2 (1) = 1.34, p = .247	χ^2 (1) = .005, p = .945	χ^2 (1) = 1.30, p = .253	-	-
Infarct size (mm^3), mean (SD)	11051 (13552)	15077 (8576)	7834 (8981)	22909 (16597)	10601 (10609)
Statistics	t(75) = .094,<br>p = .925,<br>CI [-8402, 7759]	t(75) = -1.11,<br>p = .269,<br>CI [-10092, 1624]	t(75) = 1.03,<br>p = .306,<br>CI [-2247, 10295]	t(75) = -2.00,<br>p = .049,<br>CI [-26910, 9527]	t(75) = .104,<br>p = .918,<br>CI [-11927, 10290]
Lacunes, n (%)<br>Present (31)<br>Absent (118)	 8 (26%)<br>16 (14%)	 7 (23%)<br>14 (12%)	 3 (10%)<br>14 (12%)	 3 (10%)<br>3 (3%)	 2 (7%)<br>3 (3%)
Statistics	-	-	-	-	-
CAA, n (%)<br>Present (74)<br>Absent (75)	 9 (12%)<br>15 (20%)	 8 (11%)<br>13 (17%)	 8 (11%)<br>9 (12%)	 5 (7%)<br>1 (1%)	 1 (1%)<br>4 (5%)
Statistics	χ^2 (1) = 1.69, p = .193	χ^2 (1) = 1.31, p = .253	χ^2 (1) = .052, p = .819	-	-
SVD, n (%)<br>Present (97)<br>Absent (52)	 12 (12%)<br>12 (23%)	 13 (13%)<br>8 (15%)	 13 (13%)<br>4 (8%)	 2 (2%)<br>4 (8%)	 3 (3%)<br>2 (4%)
Statistics	χ^2 (1) = 2.87, p = .090	χ^2 (1) = .110, p = .740	χ^2 (1) = 1.09, p = .296	-	-

- Chi-square expected frequency assumption violated. CAA = cerebral amyloid angiopathy, SVD = small vessel disease

Supplementary Table 3. Multinomial logistic regression statistics - SVD

		95% CI for Odds Ratio			
**None vs. Reference category – Severe SVD**					
	b (SE)	**Lower**	**Odds Ratio**	**Upper**	**P value**
**AH Medicated**	**2.6 (0.8)**	**3.1**	**14.4**	**66.1**	**.001**
Normotensive	0.2 (0.7)	0.3	1.3	4.7	.737
A0 or A1	0.9 (1.2)	0.2	2.6	28.3	.445
B0 or B1	-0.04 (0.6)	0.3	1.0	3.3	.953
Age	0.1 (0.1)	1.0	1.1	1.2	.313
Sex (Male)	-0.6 (0.6)	0.2	0.6	1.7	.303
Postmortem delay	-0.0 (0.0)	1.0	1.0	1.0	.595
Intercept	-4.5 (4.5)				.317
**Mild SVD vs. Reference category – Severe SVD**					
**AH Medicated**	**1.7 (0.8)**	**1.2**	**5.6**	**24.9**	**.025**
Normotensive	-0.7 (0.6)	0.1	0.5	1.7	.250
A0 or A1	1.4 (1.2)	0.4	4.1	43.9	.237
B0 or B1	-0.6 (0.7)	0.1	0.5	2.0	.348
Age	-0.0 (0.1)	0.9	1.0	1.1	.837
Sex (male)	0.1 (0.6)	0.4	1.1	3.4	.830
Postmortem delay	-0.0 (0.0)	1.0	1.0	1.0	.376
Intercept	1.7 (4.6)				.714
**Moderate SVD vs. Reference category – Severe SVD**					
**Medicated**	**2.6 (0.9)**	**2.5**	**13.4**	**71.7**	**.002**
Normotensive	-0.1 (0.8)	0.2	0.9	4.0	.903
A0 or A1	0.7 (1.3)	0.2	2.1	28.5	.575
B0 or B1	-0.5 (0.7)	0.1	0.6	2.6	.497
Age	-0.0 (0.1)	0.9	1.0	1.1	.987
Sex (male)	-0.1 (0.6)	0.3	0.9	3.1	.849
Postmortem delay	-0.0 (0.0)	1.0	1.0	1.0	.442
Intercept	-0.4 (5.2)				.938

Note- R^2 = .235 (Cox & Snell), .252 (Nagelkerke). Model $\chi^2(21)$ = 38.792, p = .010

Supplementary Table 4. SVD severity and AD severity score corelations

		Rarefaction Rating	PVS %	A Score (Aβ plaque stage)	B Score (NFT stage)
Rarefaction Rating	Correlation co-efficient	1			
	Sig.	-			
	n	118			
PVS %	Correlation co-efficient	**.415**	1		
	Sig.	**<.001**	-		
	n	**118**	118		
A Score (Aβ plaque stage)	Correlation co-efficient	**.198**	.033	1	
	Sig.	**.034**	.723	-	
	n	**115**	115	145	
B Score (NFT stage)	Correlation co-efficient	.174	.082	**.502**	1
	Sig.	.060	.380	**<.001**	-
	n	118	118	**145**	149
